# Suppression of histone deacetylase 1 by JSL-1 attenuates the progression and metastasis of cholangiocarcinoma *via* the TPX2/Snail axis

**DOI:** 10.1038/s41419-022-04571-9

**Published:** 2022-04-09

**Authors:** Lu Xu, Weizhong Yang, Jinhui Che, Deqiang Li, Haihong Wang, Yunjiu Li, Wuyuan Zhou

**Affiliations:** 1 Department of Hepatopancreatobillary Surgery, Xuzhou City Cancer Hospital, Xuzhou, 221000 P. R. China; 2 Deparment of Hepatobillary Surgery, Xuzhou City Cancer Hospital, Xuzhou, 221000 P. R. China

**Keywords:** Cancer, Diseases

## Abstract

Histone deacetylases (HDACs) are entwined with the pathogenesis of various cancers and potentially serve as promising therapeutic targets. Herein, we intend to explore the potential role of HDAC1 inhibitor (JSL-1) in the tumorigenesis and metastasis of cholangiocarcinoma (CC) and to highlight the molecular basis of its function. As shown by bioinformatics analysis and immunohistochemical detection, high HDAC1 expression was witnessed in CC tissues relative to matched controls from patients with cholecystitis. The molecular network that HDAC1 silencing reduced the enrichment of HDAC1 and Snail on the TPX2 promoter was identified using immunoprecipitation and chromatin immunoprecipitation assays. Both short hairpin RNA (shRNA)-mediated knockdown of HDAC1 and JSL-1 treatment exhibited anti-proliferative, anti-migration and anti-invasion effects on CC cells through downregulation of TPX2. The in vivo xenograft model was developed in nude mice. Consistently, the anti-tumorigenic and anti-metastatic properties of shRNA against HDAC1 and HDAC1 inhibitor were validated in the in vivo settings. Taken together, our data supported the notion that HDAC1 inhibitor retards the initiation and development of CC via mediating the TPX2/Snail axis, highlighting the anti-tumor molecular network functioned in CC.

## Introduction

Cholangiocarcinoma (CC) has an increasing incidence globally, which occupies approximately 15% of primary malignancies in liver and 3% in gastrointestinal system [1]. CC arises from the malignant growth of the epithelial lining of the bile ducts along the biliary tree [2]. Based on the location, CC can be classified into three subtypes: intrahepatic CC, perihilar CC and distal CC [3]. CC has an increasing global incidence and due to its silent and asymptomatic nature, particularly in the early stage, combined with the high aggressiveness, intra- and inter-tumor heterogeneity as well as chemo-resistance, the efficacy of current therapeutic therapies has been compromised, causing a poor prognosis [4]. This suggests an urgent requirement of developing more effective diagnostic and therapeutic strategies.

Known as enzymes involved in chromatin remodeling by deacetylating the lysine residues, histone deacetylases (HDACs) have been implicated in the pathogenesis of various cancers and function as novel potential therapeutic targets [5]. HDAC1 is a member of the HDACs family and can promote the tumor angiogenesis and the resultant colorectal cancer tumorigenesis [6]. Of note, knockdown of HDAC1 by HDAC inhibition retards the progression of pancreatic cancer [7]. HDAC1 has also been reported as a promising molecular target for gallbladder cancer (GBC) due to its properties of triggering the migration and invasion of GBC cells [8]. Meanwhile, HDAC1 is strongly associated with higher stage cancer, lymph node metastasis, and vascular invasion, and its overexpression indicates poor prognosis in patients with intrahepatic CC [9].

Targeting protein for Xenopus kinesin-like protein 2 (TPX2) also functions as an oncogene in different human cancers. For instance, TPX2 could be a potential target for the treatment of hepatocellular carcinoma (HCC) due to its promotive role in HCC growth [10]. Moreover, TPX2 stimulates gastric cancer cell migrative and invasive capacity [11]. On the contrary, knockdown of TPX2 results in inhibition of cell cycle arrest and proliferation of bladder cancer cells [12]. Additionally, Snail helps to induce epithelial-mesenchymal transition and immunosuppression in CC that is a highly malignant bile duct cancer, thus representing a potential therapeutic target for CC [13]. Recently, another histone demethylase lysine demethylase 3 A (also named JMJD1A) has been demonstrated to promote the progression of prostate cancer by transcriptionally activating Snail [14]. Hence, here we intended to investigate the potential implications of the HDAC1/TPX2/Snail in the pathophysiology of CC by conducting in vitro and in vivo analyses for a comprehensive understanding of tumorigenic mechanisms. JSL-1 which is characterized as an inhibitor of Class I HDACs has been highlighted as a therapeutic agent for preventing uveal melanoma [15]. We therefore not only determined the function of short hairpin RNA (shRNA)-mediated knockdown of HDAC1 but also analyzed that of HDAC1 inhibitor (JSL-1) in the progression and metastasis of CC.

## Materials and methods

### Bioinformatics analysis

CC-related dataset GSE141511 was retrieved from the Gene Expression Omnibus (GEO) database, which is composed of three control samples and three CC samples. FastQC software was used for data quality control. HISAT2 software was adopted to perform sequence alignment analysis to calculate gene counts. Significantly upregulated genes in CC samples were identified using edgeR software package in R environment with logFoldChange >1 and *p* value < 0.05 as the cutoff value. Gene Ontology (GO) and Kyoto Encyclopedia of Genes and Genomes (KEGG) analysis was performed on differentially expressed genes with regard to the involved biological process, cell component, molecular function and the mediated signaling pathways using “ClusterProfiler” package of R language. The interaction between protein coded by genes was analyzed by STRING database. Combined with online database ChIP-Alta and hTFtarget for prediction on downstream target genes, co-expression relation between transcription factors and target genes was analyzed by Gene Expression Profiling Interactive Analysis (GEPIA).

### Sample collection

The tumor tissues were surgically resected from 65 patients with CC (47 males and 18 females; aged 35–79 years with a mean age of 53.28 ± 12.74 years) at Xuzhou City Cancer Hospital between June 2016 and June 2018 between June 2016 and June 2018. Additionally, 35 patients with cholecystitis (18 males and 17 females; aged 32–56 years) surgically treated in our hospital during the same period were also recruited in this study as controls. Before experimentation, none of the enrolled patients received anti-tumor treatment. All the patients received radical cholecystectomy based on preoperative imaging and the postoperative pathological tissue sections were confirmed as CC. Patients with distant metastasis and cachexia were excluded from this study. The patients in the control group and the CC group were age-matched and gender-matched at a ratio of 1:3. The patients in the control group were diagnosed with gallstone combined with chronic cholecystitis and hospitalized at our hospital at the same time as the enrolled patients with CC and the tissue specimens were obtained by cholecystectomy.

### Immunohistochemistry

Paraffin-embedded CC tissues were sliced into 4-μm-thick sections, which were heated overnight in an oven at 37 °C, cleared three times with xylene, and rehydrated with alcohol (95%, 85%, and 75%) for 3 min. The sections were then subjected to citrate antigen retrieval and immersed in 3% H_2_O_2_ for 10 min followed by washing with phosphate-buffered saline (PBS) three times. Subsequently, the sections were blocked with 0.5% bovine serum albumin for 20 min and incubated overnight at 4 °C with the primary antibody in a wet box. After three PBS washes (3 min/time), the sections were incubated with secondary antibody at room temperature for 1 h. Afterwards, the sections were colored with diaminobenzidine and sealed with neutral balsam. At last, the results were observed in five randomly selected visual fields from each section under an inverted microscope, with 100 cells counted in each field.

### Cell culture

Human intrahepatic biliary epithelial cell line HIBEC and three CC cell lines (TFK-1, EGI-1 and HUCCT-1) (purchased from the Cell Bank of the Chinese Academy of Sciences, Shanghai, China) were cultured in Roswell Park Memorial Institute (RPMI) 1640 medium (Gibco, Grand Island, NY) containing 10% fetal bovine serum (FBS; Gibco), 10 μg/mL streptomycin, and 100 U/mL penicillin in a 5% CO_2_ incubator (Thermo Fisher Scientific, Rockford, IL) at 37 °C. CC cell line EGI-1 was cultured in minimum essential medium (MEM) containing 10% FBS, 2 × MEM amino acid and 1 mM pyruvate sodium.

### Construction of stably-infected cell line

The 293 T cells at the logarithmic growth phase were trypsinized and seeded into a 10 cm dish at a density of 5 × 10^5^ cells. After adherence to the wall for 24 h, the culture solution was renewed with preheated complete Dulbecco’s modified Eagle’s medium (DMEM), 8 mL per dish. Thereafter, a sterile 1.5 mL Eppendorf tube was added with 1 mL of Opti-MEM, 3 μg of pCMV-dR8.2 or 3 μg pCMV-VSVG packaging plasmids, and 6 μg of scramble sequence or shRNAs targeting HDAC1 (sh-HDAC1#1 and sh-HDAC1#2), for 5 min of incubation at room temperature. The tube was then added with 36 μL polyethylenimine and incubated at room temperature for 15 min. The Opti-MEM containing plasmids was then added to 293 T cells. After 12 h-culture, the medium was changed with pre-heated complete DMEM, 8 mL per dish. After 48 h, the lentivirus-containing solution was collected and filtered through a 0.4-μm filter, namely 48-h lentivirus. After 24 h of culture with 8 mL preheated complete DMEM, the lentivirus-containing solution was collected and filtered through a 0.4-μm filter head to remove cell debris, namely 72-h lentivirus which was mixed with the 48-h lentivirus. Cells were seeded into six-well plates at a density of 2 × 10^5^ cells/well, with a total of 3 parallel wells. Each well was added with 2 mL lentivirus solution and 2 mL complete culture medium, after which 8 ng/mL Polybrene was added. After 4 h of culture, the culture medium was renewed with a pre-heated complete medium. After 48 h, 1 μg/mL Puromycin was added to screen and develop a stably-infected cell line.

### RNA isolation and quantitation

Total RNA content was extracted using TRIzol reagent (15596026, Invitrogen). The extracted RNA was then reversely transcribed into complementary DNA (cDNA) using a PrimeScript RT reagent Kit (RR047A, Takara, Japan). Reverse transcription quantitative polymerase chain reaction (RT-qPCR) was conducted using a Fast SYBR Green master mix (Applied Biosystems) on an ABI PRISM 7300 System (Applied Biosystems). All investigations involved 3 parallel wells, each repeated in triplicate. Glyceraldehyde-3-phosphate dehydrogenase (GAPDH) was regarded as the internal reference and the fold changes were calculated using relative quantification (the 2^−△△Ct^ method). The synthesized primer sequences are listed in Supplementary Table 1.

### Western blot analysis

Total protein was extracted from cells and then lysed using radio-immunoprecipitation assay lysis buffer (Boster Biological Technology Co., Ltd., Wuhan, Hubei, China) containing protease inhibitor on ice for 30 min. A bicinchoninic acid assay kit (Boster) was employed to determine the protein concentration. The protein was subsequently separated using sodium dodecyl sulfate (SDS)-polyacrylamide gel electrophoresis and transferred onto nitrocellulose membranes. The membrane was blocked with 5% skimmed milk at indoor temperature for 1 h to block nonspecific binding and probed overnight at 4 °C with diluted primary antibodies against HDAC1 (ab109411, 1:1000, Abcam Inc., Cambridge, UK), Snail (ab216374, 1:1000, Abcam), TPX2 (ab252944, 1:1000; Abcam). After washing, the membrane was re-probed at indoor temperature with horseradish peroxidase-labeled secondary goat anti-rabbit immunoglobulin G (IgG) (ab205719, 1: 2000, Abcam Inc., Cambridge, UK) for 1 h. The immunocomplexes on the membrane were visualized using enhanced chemiluminescence reagent (EMD Millipore, Billerica, MA) and band intensities were quantified using the ImageJ software with β-actin serving as a loading control.

### Trypan blue staining

The cells to be tested were seeded into 24-well plates with 21 parallel wells and cells in three parallel wells were detached every day. Afterwards, 100 μL cell suspension was mixed with 100 μL trypan blue while 10 μL mixture was harvested for trypan blue counting, seven days in total.

### Colony formation assay

The cells were plated into 24-well plates (500 μL per well) with the mixture of FBS, 2 × RPMI 1640 medium and 1% agar at a ratio of 1:5:4 at 4 °C for coagulation. The cells were trypsinized and placed into centrifuge tubes for 5-min centrifugation at 1500 rpm. After supernatant removal, cells were suspended in 2 × RPMI 1640 medium without serum after which 5000 cells were extracted and water-bathed with 300 μL of the mixture of FBS, 2 × RPMI 1640 medium and 0.5% agar at 37 °C. The mixture containing cells was added to the upper layer of the 24-well plate covered with agarose and incubated at 37 °C for 14 days. Finally, the number of clones (>50 cells) was counted following observation under an inverted microscope.

### MTT assay

The cell viability was evaluated using the MTT kit (M1020; Solarbio, Beijing, China). The 100 μL of 1 × 10^3^ cells were seeded into a 96-well plate for 24-h incubation, followed by 4-h incubation with 20 μL of MTT (5 mg/mL) reagent. After removing the medium, 150 μL of dimethyl sulfoxide was added to each well. Then, a microplate reader (model 680; Bio-199 Rad, Hercules, CA) was used to measure the optical density at 490 nm.

### Scratch test

Approximately 5 × 10^5^ cells/well were added into the 6-well plate and incubated overnight in a medium containing 10% FBS. A sterile 200 μL pipette tip was utilized to scratch the bottom of the plate vertically, after which the culture medium was removed. Subsequently, 2 mL of serum-free medium was added to each well for further culture. The scratch distance was measured and photographed under an optical microscope at 0 h and 24 h, respectively to observe the cell migration.

### Transwell assay

Totally, 1 × 10^5^ cells were resuspended in 200 μL serum-free medium and added to the upper Transwell chamber and 500 μL RPMI 1640 medium containing 20% FBS was added to the lower Transwell chamber as chemokines. The loaded Transwell chamber was incubated in a 37 °C incubator for 24 h. The paraformaldehyde-fixed upper chamber was stained with 1% crystal violet. The cells not passing through the chamber were wiped off, followed by photographing and counting of the migrated cells. For invasion assay, Matrigel was prepared by mixing with medium at a ratio of 1:4 in advance. Then, 50 μL of the diluted Matrigel was placed onto the Transwell chamber, followed by 24 h of incubation in at 37 °C. Thereafter, 200 μL of serum-free medium containing 1 × 10^5^ cells was added to the upper layer of Matrigel in the chamber. The remaining procedures were similar to the cell migration experiment.

### Chromatin immunoprecipitation (ChIP)

ChIP assay was performed using an EZ-Magna ChIP TMA kit (EMD Millipore). In brief, 37% formaldehyde solution (225 μL) was added to a 10-cm culture dish containing 10 mL RPMI 1640 medium for cross-linking, which was then halted by 5 min of incubation with 1 mL of 1.375 N glycine. Cells were resuspended in SDS lysis buffer containing proteasome inhibitor and then subjected to ultrasonication to produce 200–1000 bp chromatin fragments. Afterwards, a total of 100 μL DNA fragments were mixed with 900 μL dilution buffer with 4.5 μL Protease Inhibitor Cocktail II and 60 μL Protein G Agarose over gentle shaking, followed by 1 h of incubation at 4 °C to remove non-specific binding. The mixture was centrifuged at 4000 × g and 4 °C for 1 min. The supernatant was then collected, 10 μL of which was taken and served as Input. The remaining supernatant was incubated overnight at 4 °C with 1.0 μg antibody [histone H3 lysine 9 acetylation (H3K9ac) or IgG]. Subsequently, each Input tube was eluted using 200 μL elution buffer and the antibody tube was eluted using 100 μL elution buffer. After de-crosslinking, the DNA in the complex was collected and quantified using qPCR on a CFX 96 Real-Time system (Bio-Rad).

### Co-immunoprecipitation (Co-IP) assay

PBS-washed cells were subjected to 5-min centrifugation at 3000 r/min and 4 °C. After removing the supernatant, the precipitate was fully lysed on ice using E1A lysis buffer supplemented with protease inhibitor cocktail (Roche), followed by 3% power ultrasound for 3 min and centrifugation (4 °C, 12000 r/min) for 15 min. Then, 40 μL of the lysate was taken as the IB control group (Lysate). The remaining (about 650 μL) was incubated with aforementioned antibodies for 3 h at 4 °C and 40 μL protein A/G-agarose at 4 °C for more than 8 h. After washing with 1 mL E1A lysis buffer, the lysate was cooked at 100 °C for 15 min with E1A lysis buffer an equal amount of 2 × sample buffer, followed by Western blot analysis.

### Nude mouse tumor xenograft model and tumor metastasis model

A total of 1 × 10^5^ TFK-1 cells were resuspended in 200 μL PBS and inoculated subcutaneously into female nude mice (aged 4–6 weeks). About three weeks later, the subcutaneous tumors were tangible. After that, the length (a) and width (b) of the tumor were measured with an electronic vernier caliper every other day and the tumor volume (V) was calculated using the formula: V = a^2^ × b × 0.4. When the tumor volume reached about 100 mm^3^, the nude mice were randomly assigned into the control group (*n* = 6; nude mice injected with placebo intraperitoneally) and the JSL-1 group (*n* = 6; nude mice injected with 50 mg/kg JSL-1 intraperitoneally) or the scramble group and the sh-HDAC1 group. After two weeks, the nude mice were euthanized and the subcutaneous tumor was extracted, weighed, and photographed. The proteins were isolated from tumor tissues for Western blot analysis and fresh tissues were fixed with formalin for hematoxylin-eosin (HE) staining and immunohistochemistry.

In brief, 1 × 10^5^ cells were resuspended in 200 μL serum-free medium and injected into the spleen of 4 to 6-week-old nude mice. After about four weeks, the nude mice were euthanized and the liver was excised immediately. The nodules on the liver surface were counted. The liver tissues were fixed and subjected to HE staining.

### Statistical analysis

Statistical analysis of data was carried out using SPSS 21.0 software (IBM Corp., Armonk, NY). All measurement data were summarized as mean ± standard deviation. If data obeying normal distribution and homogeneity of variance, comparisons between two groups were conducted using unpaired *t-*test. Data among multiple groups were compared by one-way analysis of variance (ANOVA) with Tukey’s post-hoc test. Repeated measures ANOVA with Bonferroni post hoc test was applied for the comparison of tumor volume at different time points. Two-way ANOVA was applied for comparison of cell viability at different time points. Spearman correlation analysis was used for the comparison of ordinal data between two groups. *p* < 0.05 represents a statistically significant difference.

## Results

### Bioinformatics analysis predicts that HDAC1 transcription activates TPX2 in CC progression

Analysis of the GSE141511 dataset yielded 3,370 significantly upregulated genes and 4,169 significantly downregulated genes in CC samples (Fig. S1A). Further GO and KEGG analysis revealed organic anion transport, small GTPase mediated signal transduction and positive regulation of proteolysis as main involved biological process, actin cytoskeleton and transcription regulator complex as cell component while active transmembrane transporter activity as molecular function (Fig. S1B) as well as phosphoinositide dependent kinase-1-Akt signaling pathway (Fig. S1C). In addition, 124 genes were involved in transcription regulator complex and protein-protein interaction network was plotted based on STRING database (Fig. S1D), revealing HDAC1 as the core position (Degree = 40).

Analysis of the GSE141511 dataset showed that HDAC1 was significantly highly expressed in CC (Fig. S1E). Online database ChIP-Alta and hTFtarget predicted TPX2 as the downstream target gene of transcription factor HDAC1 along with the binding sites (Fig. S1F). By means of GEPIA, HDAC1 and TPX2 were positively correlated in CC based on Pearson’s correlation coefficient (Fig. S1G). Taken together, HDAC1 transcription was predicted to activate TPX2 in CC progression.

### Upregulation of HDAC1 in CC clinical tissues and cells

**Fig. 1:**
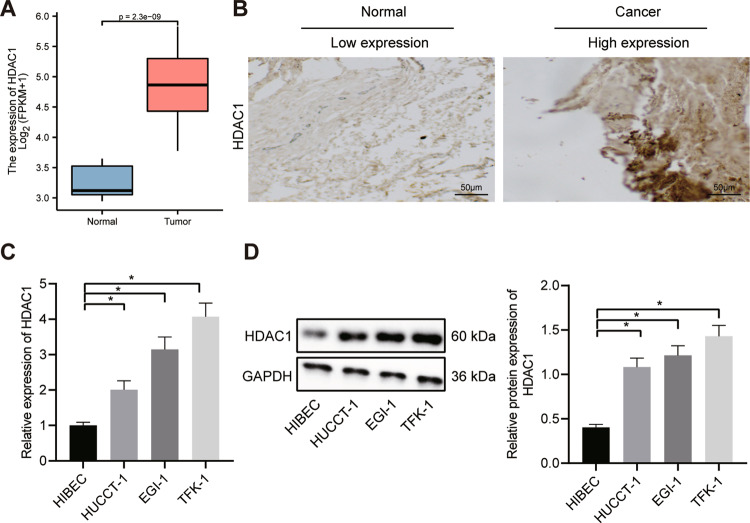
HDAC1 is upregulated in CC clinical samples and cells. **A** TCGA database analysis of HDAC1 protein in CC tissues (*n* = 36) and control tissues (*n* = 9). **B** The immunohistochemical staining of HDAC1 protein in CC tissues (*n* = 65) and normal bile duct tissues (*n* = 35). **C** HDAC1 mRNA expression in TFK-1, HUCCT-1, EGI-1 and HIBEC cell lines determined by RT-qPCR. D, HDAC1 protein expression in TFK-1, HUCCT-1, EGI-1 and HIBEC cell lines measured by Western blot analysis. *p < 0.05. In panels (**A**) and (**B**), data were compared by unpaired *t-*test. In panels (**C**) and (**D**), data were compared by one-way ANOVA with Tukey’s post hoc test. The cell experiment was independently repeated three times.

Initially, TCGA database analysis revealed upregulated expression of HDAC1 in CC tissues (*n* = 36), as compared to normal bile duct tissue (*n* = 9) (Fig. 1A). Then, we examined the expression of HDAC1 in CC tissues (*n* = 65) using immunohistochemistry. Compared with normal tissues (*n* = 35), HDAC1 expression was elevated in CC tissues (Fig. 1B).

Meanwhile, RT-qPCR and Western blot analysis results showed that HDAC1 expression was much higher in CC cell lines (TFK-1, HUCCT-1 and EGI-1) than that in HIBEC cells. TFK-1 cell line presented the highest HDAC1 expression among the CC cell lines, which was selected for subsequent experiments (Fig. 1C, D). Taken together, these results indicated the upregulated expression of HDAC1 in both CC tissues and cells, suggesting the potential involvement of HDAC1 in the pathogenesis of CC.

### Silencing of HDAC1 represses the proliferation and tumorigenicity of CC cells in vivo and in vitro

**Fig. 2:**
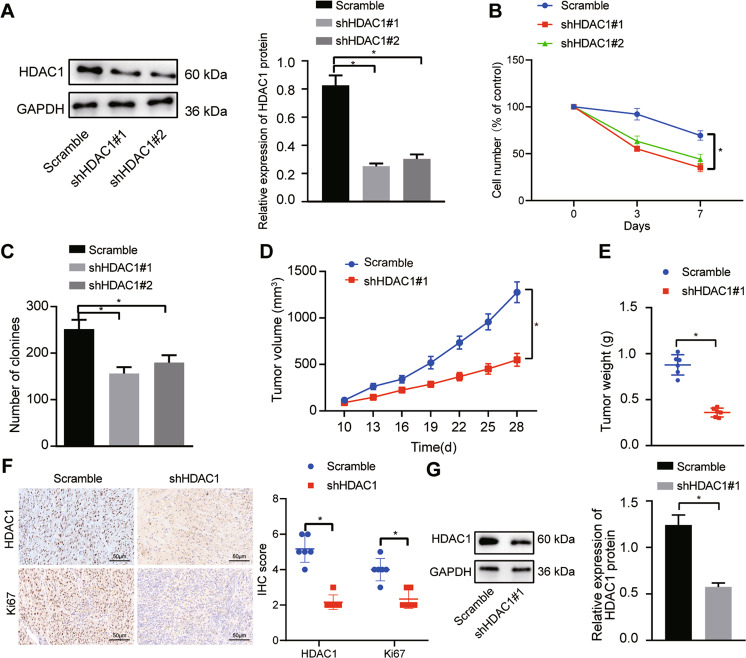
Downregulation of HDAC1 represses the growth of CC cells in vivo and in vitro. **A** Knockdown efficiency of shHDAC1#1 and shHDAC1#2 in TFK-1 cells detected by Western blot analysis. **B** Proliferation of TFK-1 cells upon HDAC1 silencing assessed by trypan blue staining. **C** The number of newly formed colonies in TFK-1 cells upon HDAC1 silencing tested by colony formation assay. **D** Tumor volume in nude mice in response to HDAC1 silencing. **E** Tumor weight in nude mice in response to HDAC1 silencing. **F** Immunohistochemical analysis of Ki67 and HDAC1 proteins in the tumor tissues of nude mice in response to HDAC1 silencing. **G** HDAC1 protein expression in tumor tissues of nude mice in response to HDAC1 silencing measured by Western blot analysis. *p < 0.05. In panels (**A**) and (**C**), data were compared by one-way ANOVA with Tukey’s post hoc test. In panels (**E**–**G**), data were compared by unpaired *t*-test. In panels (**B**) and (**D**), data were compared by repeated measures ANOVA with Bonferroni post hoc test. The cell experiment was independently repeated three times. *n* = 6 for animal experiment.

For further investigation on effect of HDAC1 on CC cell growth, HDAC1 was silenced in TFK-1 cell line. Western blot analysis confirmed the knockdown efficiency of HDAC1 in TFK-1 cells. As shown in Fig. 2A, HDAC1 expression was reduced in TFK-1 cells upon treatment with sh-HDAC1#1 and sh-HDAC1#2, of which sh-HDAC1#1 revealed higher silencing efficiency and was therefore selected for subsequent experiments.

The results of trypan blue staining and colony formation assays revealed that HDAC1 silencing inhibited the proliferation of TFK-1 cells (Fig. 2B) and decreased the number of newly formed colonies (Fig. 2C), suggesting the inhibitory effect of silencing HDAC1 on CC cell proliferation in vitro.

We further aimed to demonstrate whether HDAC1 silencing could inhibit the tumorigenic capability of CC cells in an in vivo tumor formation model. The in vivo experimental data illustrated that HDAC1 silencing led to reductions in tumor volume, tumor size, and weight in nude mice (Fig. 2D, E). Additionally, the results of immunohistochemistry and Western blot analysis displayed that HDAC1 knockdown significantly decreased the expression of cell proliferation antigen Ki67 as well as HDAC1 (Fig. 2F, G). Therefore, HDAC1 silencing could inhibit the proliferation of CC cells both in vivo and in vitro.

### Silencing of HDAC1 impedes the migratory, invasive and metastatic potentials of CC cells in vivo and in vitro

**Fig. 3:**
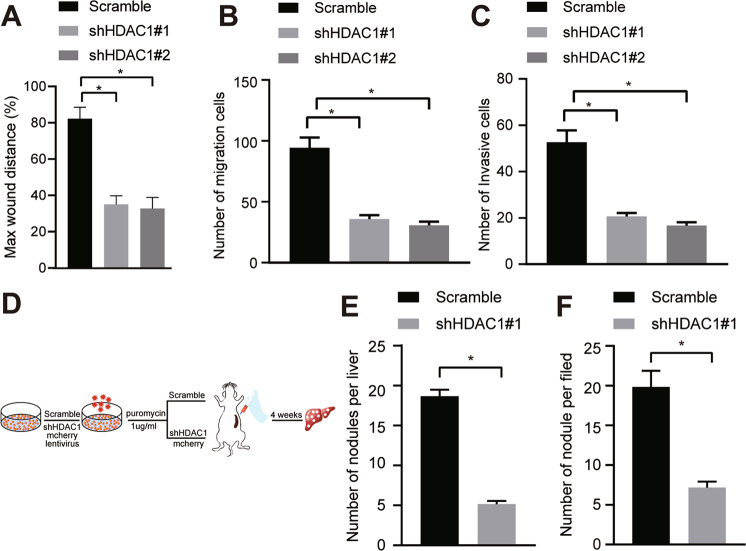
Loss of HDAC1 represses CC cell migration and invasion in vivo and in vitro. **A** Wound healing ability of TFK-1 cells upon HDAC1 silencing evaluated by scratch test. **B** TFK-1 cell migration upon HDAC1 silencing assessed by Transwell assay. **C** TFK-1 cell invasion upon HDAC1 silencing tested by Transwell assay. **D** Program flowchart of tumor metastasis model developed in nude mice and HDAC1 silencing. **E** The number of nodules visualized on the liver surface in tumor-bearing nude mice in response to HDAC1 silencing. **F** Analysis for HE staining of nodules in the liver of tumor-bearing nude mice in response to HDAC1 silencing. *p < 0.05. In panels (**A**–**D**), data were compared by one-way ANOVA with Tukey’s post hoc test. In panels (**E**) and (**F**), data were compared by unpaired *t*-test. The cell experiment was independently repeated three times. *n* = 6 for animal experiment.

The next focus of this study was to elucidate the effect of HDAC1 on CC cell migration and invasion in vitro using Scratch test and Transwell migration and invasion assays. The migration (Fig. 3A, B, Fig. S2A,B) and invasion (Fig. 3C, Fig. S2C) abilities of TFK-1 cells were diminished upon HDAC1 silencing.

Furthermore, an in vivo tumor metastasis model was developed to testify the effect of HDAC1 on tumor metastasis. The mCherry-labeled TFK-1 cells were inoculated into the spleen of nude mice for observation on liver metastasis and number of nodules 4 weeks later. As shown by bioluminescence imaging and HE staining, HDAC1 silencing resulted in fewer nodules visualized on the liver surface and nodules formed in the liver of tumor-bearing nude mice 4 weeks later (Fig. 3D–F). Collectively, silencing HDAC1 prevented migration and invasion abilities of HBC cells both in vitro and in vivo from metastasis.

### HDAC1 promoted the progression of CC by upregulating TPX2

**Fig. 4:**
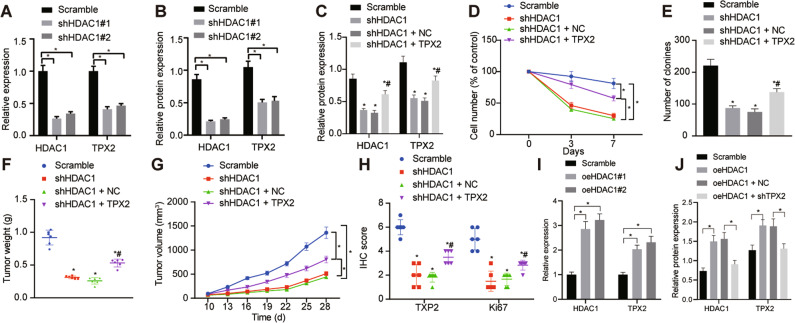
HDAC1 loss-of-function represses the growth of CC cells *via* suppression of TPX2. **A** TPX2 expression upon HDAC1 silencing determined by RT-qPCR. **B** TPX2 protein expression in TFK-1 cells upon HDAC1 silencing measured by Western blot analysis. **C** The transfection efficiency of sh-HDAC1 and oe-TPX2 in TFK-1 cells tested by Western blot analysis. **D** Proliferation of TFK-1 cells shown by trypan blue staining after HDAC1 silencing alone or with TPX2 overexpression in combination. **E** The number of newly formed colonies in TFK-1 cells tested by colony formation assay after HDAC1 silencing alone or with TPX2 overexpression in combination. **F**, **G** Quantitative analysis of xenografted tumor volume (**F**) and weight (**G**) after HDAC1 silencing alone or with TPX2 overexpression in combination. **H** Immunohistochemical detection of Ki67, TPX2 and HDAC1 proteins in tumor tissues of nude mice after HDAC1 silencing alone or with TPX2 overexpression in combination. I, TPX2 mRNA expression in TFK-1 cells overexpressing HDAC1 measured by RT-qPCR. J, TPX2 and HDAC1 protein expression in TFK-1 cells overexpressing HDAC1 or combined with TPX2 silencing measured by Western blot analysis. *p < 0.05 versus the Sceamble group. # *p* < 0.05 versus the shHDAC1 or the shHDAC1 + NC group. In panels (**A**–**D**), (**F**–**H**) and (**J**), data were compared by one-way ANOVA with Tukey’s post hoc test. In panels (**E**) and (**I**), data were compared by repeated measures ANOVA with Bonferroni post hoc test. The cell experiment was independently repeated three times. *n* = 6 for animal experiment.

RT-qPCR for determination on regulatory role of HDAC1 on TPX2 in CC revealed that HDAC1 silencing downregulated the expression of TPX2 (Fig. 4A). We then determined the resultant expression of TPX2 in TFK-1 cells treated with sh-HDAC1 by RT-qPCR and Western blot analysis. Our results showed a reduction in TPX2 mRNA and protein expression following HDAC1 knockdown (Fig. 4B, Fig. S3A).

Additionally, rescue experiments were conducted to investigate the role of HDAC1 in the development of CC through TPX2. The obtained data revealed that the reduction of TPX2 caused by HDAC1 interference was reversed by its combination with TPX2 overexpression (Fig. 4C, Fig. S3B). Consequently, TFK-1 cell proliferating ability decreased by HDAC1 silencing was partially rescued by overexpression of TPX2 as revealed from trypan blue staining and colony formation assays (Fig. 4D, E), suggesting that silence of HDAC1 downregulated TPX2 expression to suppress CC proliferation in vitro.

Meanwhile, TPX2 overexpression diminished the inhibitory effect of HDAC1 silencing on tumor volume and weight of nude mice (Fig. 4F, G). The immunohistochemical results exhibited that Ki67 expression reduced by HDAC1 silencing was neutralized by TPX2 overexpression (Fig. 4H). In addition, the results of RT-qPCR revealed that the TPX2 mRNA expression was augmented in TFK-1 cells overexpressing HDAC1 (Fig. 4I). The results of Western blot analysis showed that the promoting effect of HDAC1 overexpression on the TPX2 protein expression was reversed by further knockdown of TPX2 (Fig. 4J, Fig. S3C). Hence, the aforementioned results unraveled that downregulated HDAC1 inhibited the growth of CC cells by downregulating the expression of TPX2 in vivo.

### Silencing of HDAC1 impaired the invasive and metastatic potentials of CC cells by reducing TPX2

**Fig. 5:**
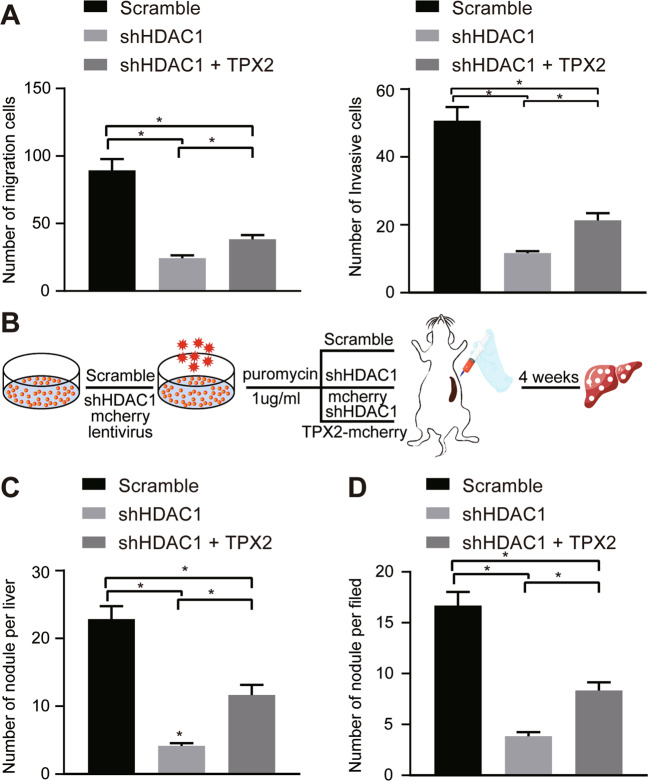
HDAC1 knockdown represses the migratory, invasive and metastatic properties of CC cells *via* suppression of TPX2. **A** Cell migration and invasion upon HDAC1 silencing alone or with TPX2 overexpression in combination assessed by Transwell assay. **B** Program flowchart of tumor metastasis model developed in nude mice and HDAC1 silencing alone or with TPX2 overexpression in combination. **C** The number of nodules on the mouse liver surface. **D** The number of nodules inside the mouse liver. *p < 0.05. Data were compared by one-way ANOVA with Tukey’s post hoc test. The cell experiment was independently repeated three times. *n* = 6 for animal experiment.

With results determining the inhibitory action on CC occurrence, CC metastasis affected by HDAC1-TPX2 was further studied. The results of the Transwell assay depicted that the TPX2 overexpression partially abrogated the suppressive effect of sh-HDAC1 on the migration and invasion of CC cells (Fig. 5A, B).

In vivo experimental results also revealed that the reduction of nodules on the liver surface and inside the liver by HDAC1 silencing was reversed by restoration of TPX2 (Fig. 5C, D). The above diagrams served to illustrate that downregulated HDAC1 restrained the tumor invasion and metastasis in CC by diminishing TPX2.

### HDAC1 knockdown downregulated the TPX2 expression by suppressing Snail

**Fig. 6:**
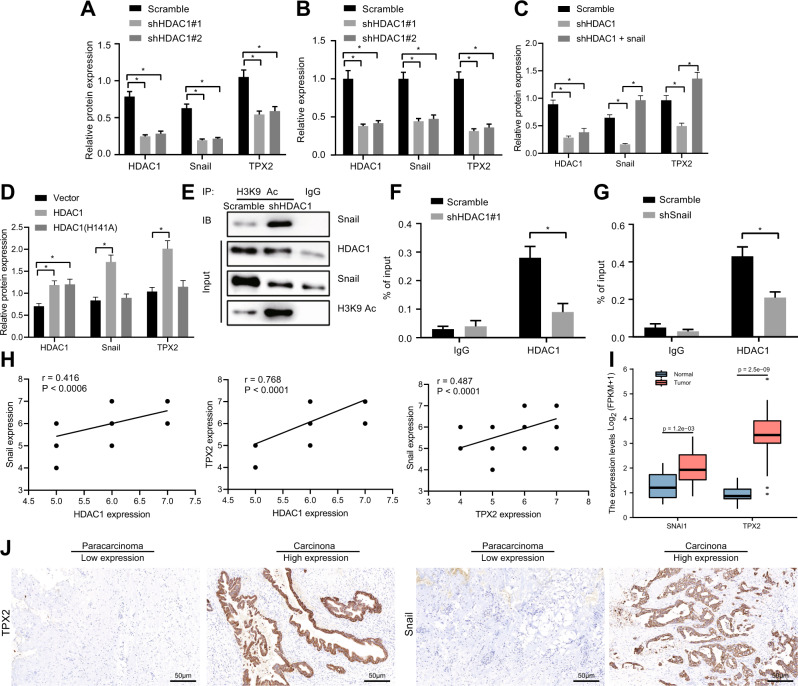
HDAC1 silencing downregulated the TPX2 expression *via* suppression of Snail. **A** The protein expression of Snail in TFK-1 cells upon HDAC1 silencing measured by Western blot analysis. **B** mRNA expression of Snail in TFK-1 cells upon HDAC1 silencing measured by RT-qPCR. **C** TPX2 protein expression in TFK-1 cells after HDAC1 silencing alone or with Snail overexpression in combination measured by Western blot analysis. **D** The expression of Snail and TPX2 proteins following HDAC1 mutation measured by Western blot analysis. **E** The interaction between Snail and H3K9 histone acetylation upon HDAC1 silencing identified by IP assay. **F** The enrichment of HDAC1 and Snail on the TPX2 promoter upon HDAC1 silencing assessed by ChIP. **G** The enrichment of HDAC1 and Snail on the TPX2 promoter upon Snail silencing assessed by ChIP. **H** Correlations of HDAC1 with TPX2 and Snail in clinical CC tissues (*n* = 65) analyzed using immunohistochemistry. **I** TCGA database analysis of Snail and TPX2 proteins in CC tissues (*n* = 36) and normal tissues (*n* = 9). **J** Immunohistochemical staining of Snail and TPX2 proteins in CC tissues (*n* = 65) and normal tissues (*n* = 35). *p < 0.05. In panels (**A**–**E**), data were compared by one-way ANOVA with Tukey’s post hoc test. In panels (**F**) and (**G**), data were compared using unpaired *t*-test. The cell experiment was independently repeated three times.

Then we further validated whether HDAC1 regulating TPX2 gene expression through Snail. The results of Western blot analysis and RT-qPCR exhibited a reduction in the mRNA and protein expression of Snail in TFK-1 cells after HDAC1 silencing (Fig. 6A, B, Fig. S3D).

In addition, the expression of TPX2 in TFK-1 cells reduced by sh-HDAC1 was rescued by co-treatment with oe-Snail (Fig. 6C, Fig. S3E), which indicated that HDAC1 regulated the expression of TPX2 by mediating the transcription factor Snail.

Next, HDAC1 was mutated in TFK-1 cells, after which TPX2 and Snail protein expression was measured by Western blot analysis. A noticeable increase was observed in the protein expression of Snail and TPX2 following overexpression of HDAC1, while HDAC1 mutation failed to increase the expression of Snail and TPX2 (Fig. 6D, Fig. S3F), suggesting that HDAC1 could indeed modulate the expression of Snail and TPX2.

As HDAC1 was a member of HDACs, we speculated that HDAC1 could mediate gene expression *via* histone acetylation. Moreover, IP results presented that HDAC1 silencing enhanced the binding of Snail to H3K9 acetylation (Fig. 6E).

The results of ChIP showed that the enrichment of HDAC1 and Snail on the TPX2 promoter was inhibited by HDAC1 silencing or Snail silencing (Fig. 6F, G). Analysis by immunohistochemical staining exhibited positive correlation between HDAC1 and TPX2 as well as between HDAC1 and Snail in CC tissues of patients (*n* = 65) (Fig. 6H, Fig. S2D). In addition, analysis of the TCGA database suggested higher expression of Snail and TPX2 in clinical tissue samples of CC patients (*n* = 36) compared with normal tissue samples (*n* = 9) (Fig. 6I). The results of immunohistochemical staining showed that the protein expression of Snail and TPX2 was increased in CC tissues (*n* = 65) (Fig. 6J). These results showed that silencing of HDAC1 downregulated the TPX2 expression by inhibiting Snail.

### HDAC1 inhibitor JSL-1 hindered the growth of CC cells via the Snail/TPX2 axis

**Fig. 7:**
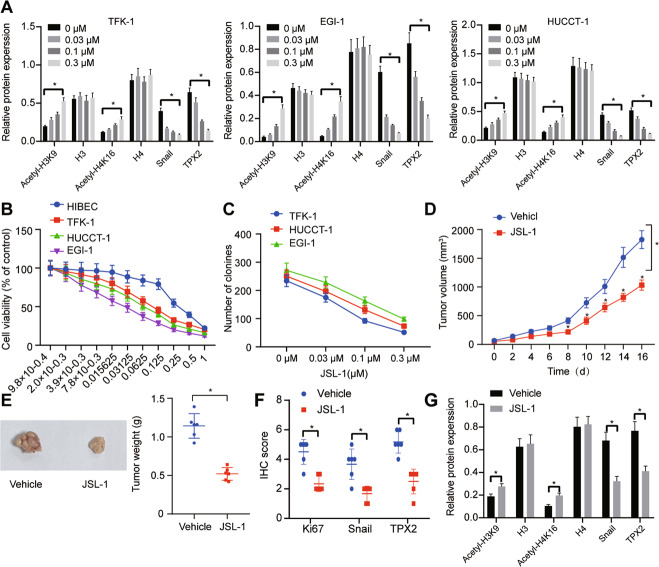
JSL-1 impedes the proliferation of CC cells and tumorigenesis *via* repression of Snail/TPX2. **A** Acetylation level of H3K9 and H4K16 and expression of Snail and TPX2 proteins in TFK-1 cells upon JSL-1 treatment. **B** Viability of TFK-1 cells upon JSL-1 treatment at different doses detected by MTT assay. **C** Number of colonies in TFK-1 cells following JSL-1 treatment assessed by colony formation assay. **D** Tumor volume of nude mice upon JSL-1 treatment. **E** Tumor weight of nude mice upon JSL-1 treatment. **F** Ki67-positive cells and positive Snail and TPX2 protein expression in tumor tissues of nude mice treated with JSL-1 detected by immunohistochemistry. **G** The expression of Snail and TPX2 proteins and acetylation level of H3K9 and H4K16 in tumor tissues of nude mice after JSL-1 treatment measured by Western blot analysis. *p < 0.05. In panel (**A**), data were compared by one-way ANOVA with Tukey’s post hoc test. In panels (**E**–**G**), data were compared using unpaired *t*-test. In panels (**B**–**D**), data were compared using repeated measures ANOVA with Bonferroni post hoc test. The cell experiment was independently repeated three times. *n* = 6 for animal experiment.

To further testify whether HDAC1 regulates the expression of TPX2 through its HDAC activity, JSL-1, the inhibitor of Class I HDACs, was utilized to block the activity of HDAC1. As depicted in Fig. 7A, Fig. S3G, JSL-1 treatment increased the acetylation level of H3K9 and H4K16, indicating that JSL-1 could markedly disrupt the role of HDAC in TFK-1 cells. JSL-1 treatment was also shown to reduce the expression of Snail and TPX2 proteins in TFK-1 cells.

Subsequent experiment offered data suggesting that JSL-1 could impede TFK-1 cell viability in a dose-dependent manner (Fig. 7B) and colony formation capability (Fig. 7C).

We then intended to clarify the antitumor effect of JSL-1 in vivo. As presented in Fig. 7D, E, tumor volume and weight of nude mice were reduced upon JSL-1 treatment. In addition, the levels of Snail, TPX2, and Ki67, as reflected by immunohistochemical scores, were curbed by JSL-1 (Fig. 7F, Fig. S2E). As revealed by the data of Western blot analysis, JSL-1 treatment in nude mice contributed to elevated acetylation level of H3K9 and H4K16 yet downregulated expression of Snail and TPX2 proteins (Fig. 7G, Fig. S3H).

The above-mentioned results unraveled that JSL-1 might affect the proliferative and tumorigenic potentials of CC cells through disrupting the Snail/TPX2 signaling axis.

### HDAC1 inhibitor JSL-1 impaired the migratory, invasive and metastatic abilities of CC cells

**Fig. 8:**
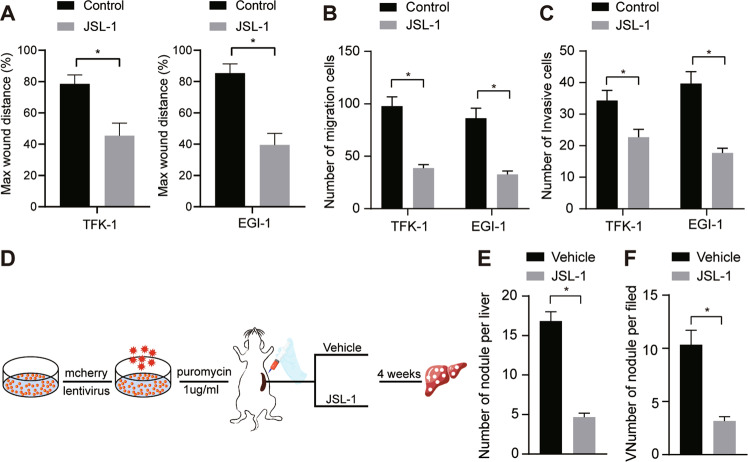
JSL-1 curbs the migratory, invasive and metastatic abilities of CC cells in vitro and in vivo. **A** Wound healing ability of cells treated with JSL-1 assayed by scratch test. **B** Cell migration upon JSL-1 treatment assessed by Transwell assay. **C** Cell invasion upon JSL-1 treatment assessed by Transwell assay. **D** Program flowchart of tumor metastasis model developed in nude mice and JSL-1 treatment. **E** The number of nodules in the mouse liver surface after JSL-1 treatment. **F** Analysis for HE staining of the nodules inside the mouse liver and quantitative analysis after JSL-1 treatment. *p < 0.05. In panels (**A**–**C**) and (**E**, **F**), data were compared by unpaired *t*-test. The cell experiment was independently repeated three times. *n* = 6 for animal experiment.

We further shifted our attention to verifying the effect of HDAC1 inhibitor on the migration, invasive, and metastatic abilities of CC cells. The results of scratch test shown in Fig. 8A, Fig. S2F presented that JSL-1 treatment inhibited the wound healing ability of CC cells. Transwell assay also revealed the inhibitory role of JSL-1 in the migratory and invasive properties of CC cells (Fig. 8B, C). Moreover, in vivo experimental results displayed that JSL-1 treatment resulted in reductions in the number of nodules on the mouse liver surface and that inside the mouse liver (Fig. 8D–F). These results collectively supported the conclusion that HDAC1 inhibitor JSL-1 could inhibit the CC invasion and metastasis in the context of in vitro and in vivo.

## Discussion

Emerging evidence has highlighted the therapeutic potential of inhibitors of HDACs in CC [16, 17]. The gathered in vivo and in vitro findings from the current study consistently unraveled that the downregulation of HDAC1 by either shRNA or JSL-1 (inhibitor of Class I HDACs) could potentially prevent the initiation and progression of CC *via* suppression of Snail/TPX2.

An overwhelming number of studies have proven that HDAC1 is tightly correlated with cancerogenesis. For example, HDAC1 has been demonstrated to be tumor promoter in a range of malignancies, covering gastrointestinal tumors such as colorectal cancer [18], and gastric cancer [19], as well as HCC [20] and ovarian cancer [21]. Inhibition of this HDAC has recently been corroborated to prevent the progression of gastric cancer [22] and esophageal carcinoma [23]. The major results from the present study revealed that HDAC1 was highly expressed in CC clinical samples and cells, whereas shRNA-induced silencing of HDAC1 contributed to prevention against CC progression, supported by the restrained proliferative, migratory and invasive capabilities of CC cells. Partially in agreement with this finding, a previous study has demonstrated that HDAC1 knockdown by lentivirus delivery of HDAC1-specific shRNA can repress the migrative and invasive abilities of GBC cells [8]. In addition, treatment with a HDAC inhibitor, namely suberoylanilide hydroxamic acid, has been substantiated to retard the proliferative and metastatic properties of GBC cells and tumor formation in GBC xenograft models [24]. Also, an anti-tumor effect of JSL-1 on uveal melanoma has been illustrated in previous studies [25, 26]. In the current study, JSL-1, an inhibitor of Class I HDACs, was shown to hinder the malignant phenotypes of CC cells both in vitro and in vivo. These findings highlighted shRNA targeting HDAC1 or JSL-1 as a potential therapeutic approach or agent for CC.

TPX2 has been shown to promote the oncogenesis in the context of several cancers such as gastric cancer, colon cancer and glioblastoma [27–29]. Inhibition of TPX2 stimulates G2-M phase-arrested cells and apoptosis while diminishing the invasive and migratory potentials of CC cells [30]. Moreover, inhibitors of HDAC possess the ability to reduce the expression of TPX2, mitotic spindle formation-related protein in glioma cells and in human glioblastoma multiforme primary cultures [31]. Our study identified that HDAC1 silencing enhanced the binding of Snail to H3K9 acetylation and JSL-1 treatment was found to inhibit the H3K9 acetylation and downregulate TPX2 expression. Knockdown of TPX2 is paralleled by a decrease in the levels of histone H4 acetylated at lysine 16 during G1-phase, showing potential implications for DNA damage response [32]. Therefore, silencing of HDAC1 potentially prevented the progression and metastasis of CC by downregulating TPX2 expression, which was validated by the rescue experiments in CC cells and murine model. However, due to the extensive regulatory network of HDAC1 [33, 34], we cannot exclude the involvement of other targets in the regulation of HDAC1 on the progression and metastasis of CC. Thus, the specific mechanism involving the HDAC1/TPX2 axis in CC needs to be identified through additional studies.

Mechanically, this study uncovered that silencing of HDAC1 disrupted the TPX2 expression by inhibiting Snail. HDAC1 knockdown has been consistently proven to decrease the expression of Snail in glioma cells thus suppressing cancer progression [35]. A histone demethylase KDM3A has been revealed to transcriptionally activate Snail expression *via* H3K9me1 and H3K9me2 demethylation at its special promoter region, consequently promoting prostate cancer progression [14]. Snail is well-known to be a prominent epithelial-mesenchymal transition transcription factor and promotes tumor invasion and metastasis in GBC [36, 37]. Deficiency of TPX2 is able to hinder the epithelial-mesenchymal transition of tumor cells [11]. Therefore, TPX2 is inferred to be in a positive correlation with Snail. Moreover, we experimentally demonstrated that HDAC1 silencing downregulated the enrichment of Snail on the TPX2 promoter. We thus reach a conclusion that downregulated HDAC1 may impede the growth of CC cells through repression of TPX2 *via* downregulating Snail. As a novel inhibitor of Class I HDACs, JSL-1 has the potential to regulate the expression of acetylation [15, 25]. In our research, we found that application of JSL-1 could reduce the expression of Snail and TPX2, which is mainly due to the fact that JSL-1 reduced the expression of HDAC1 and thus affected the expression of its downstream genes Snail and TPX2 through the interaction of histone acetylation. This highlights the innovative target of JSL-1 for drug development.

## Conclusion

**Fig. 9:**
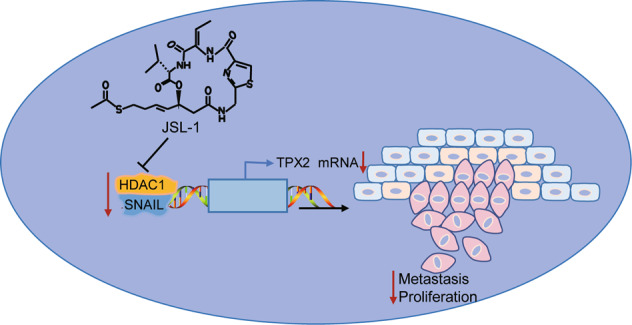
The schematic map of the regulatory network HDAC1/Snail/TPX2 in CC. JSL-1 blocks the enrichment of HDAC1 and Snail on the TPX2 promoter and then downregulates TPX2 expression by inhibiting HDAC1, thus impeding the growth and metastasis of CC.

To sum up, this study demonstrates that the suppression of HDAC1 can decelerate the tumorigenesis and tumor metastasis in CC *via* modulation of the Snail/TPX2 axis (Fig. 9). The present study reveals the critical therapeutic value of HDAC1 inhibitor JSL-1 in CC. However, it is also recommended that future studies are required for the in-depth analysis of the HDAC1/Snail/TPX2 axis in CC due to the enigmatic knowledge regarding the interaction between Snail and TPX2.

## Supplementary information

Supplementary files

Reproducibility Checklist

## Data Availability

The datasets generated and/or analysed during the current study are available in the manuscript and supplementary materials.
