# Supplementary material for: Suppression of histone deacetylase 1 by JSL-1 attenuates the progression and metastasis of cholangiocarcinoma via the TPX2/Snail axis
Source: Cell Death Dis. 2022 Apr 9;13(4):324. doi: 10.1038/s41419-022-04571-9 (PMC8993895; doi:10.1038/s41419-022-04571-9)
Supplement: Supplementary file 1 — Supplementary files [file 41419_2022_4571_MOESM1_ESM.docx]

**
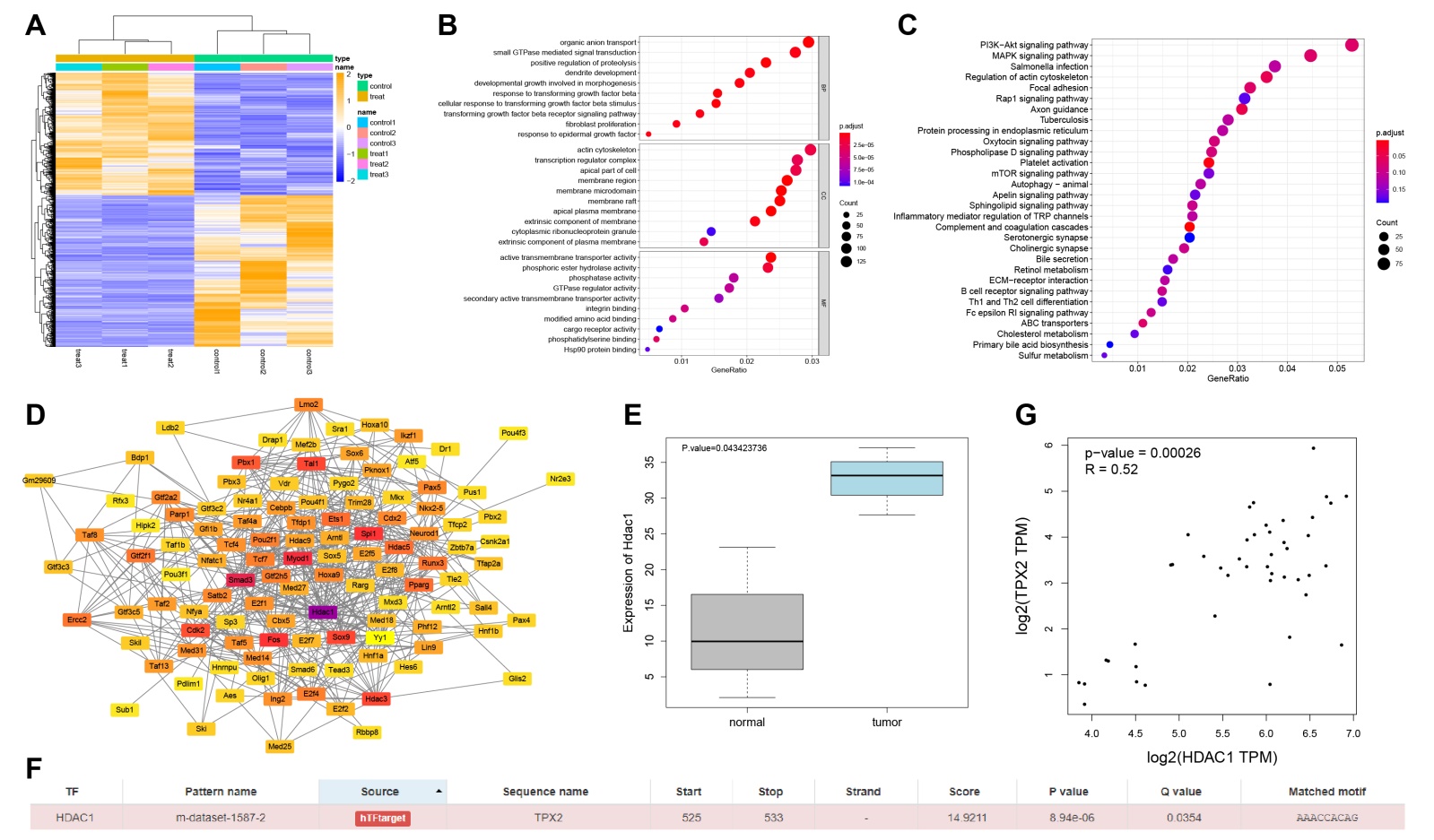
**

**Fig. S1** Bioinformatics analysis. A, A heat map of differentially expressed genes in control samples (n = 3) CC samples (n = 3) in the GSE141511 dataset. Color scale from orange to blue represents high to low expression. B, Enrichment results of biological process (BP), cell component (CC) and molecular function (MF) involving differentially expressed genes with the X-axis showing the geneRatio. C, KEGG signaling pathways involving differentially expressed genes. D, Protein-protein interaction network coded by genes. The range from purple to yellow represents high to low degree value. E, HDAC1 expression in CC samples (n = 3) and normal samples (n = 3) in the GSE141511 dataset. F, Binding sites between transcription factor HDAC1 and downstream gene TPX2. G, Co-expression relation between HDAC1 and TPX2 in CC samples from TCGA (R = 0.52, *p*-value = 0.00026).

**
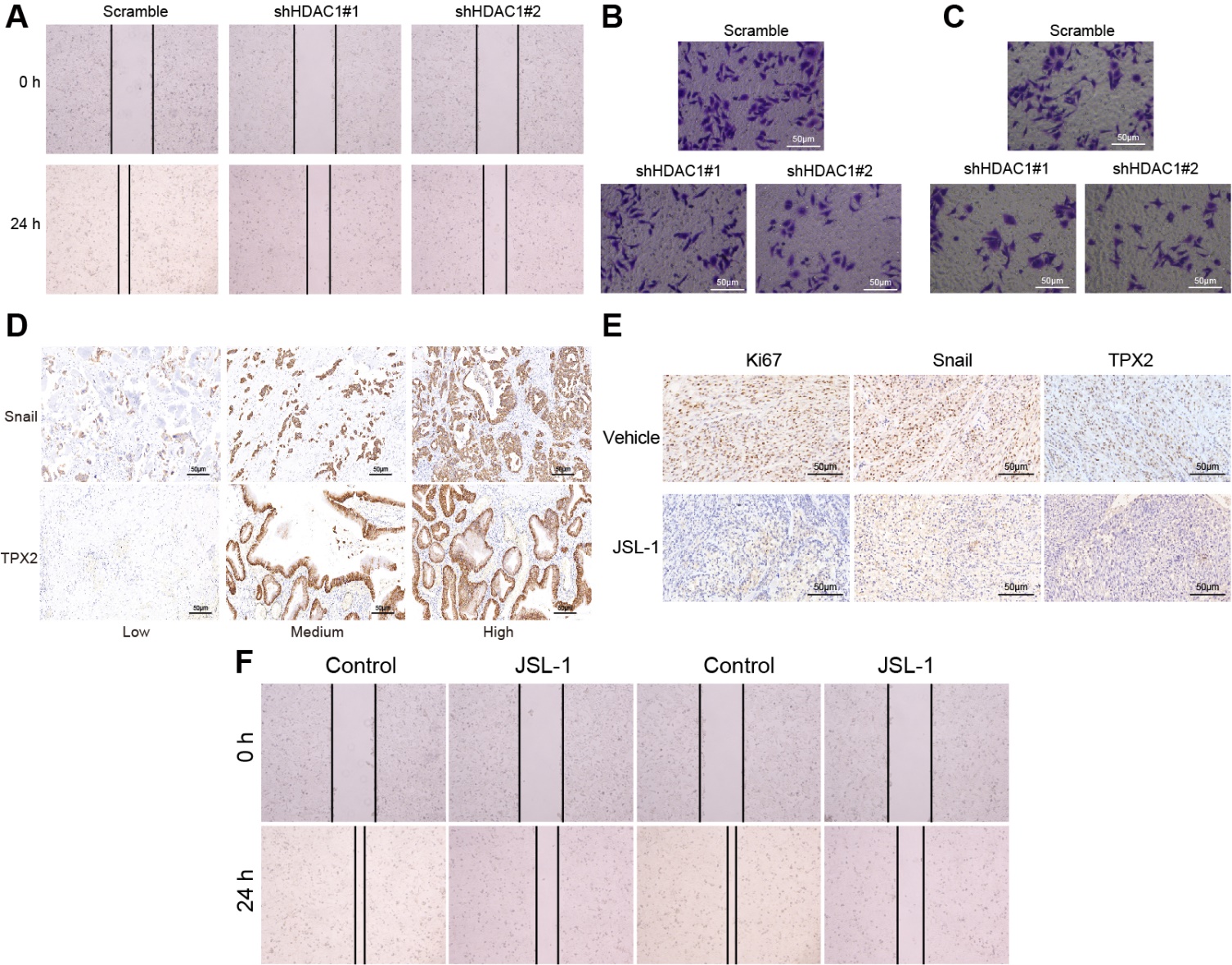
**

**Fig. S2** Representative images of scratch test (A, F), Transwell assay (B, C), and immunohistochemistry (D, E). A, Wound healing ability of TFK-1 cells upon HDAC1 silencing evaluated by scratch test (quantification in panel 3A). B, TFK-1 cell migration upon HDAC1 silencing assessed by Transwell assay (quantification in panel 3B). C, TFK-1 cell invasion upon HDAC1 silencing tested by Transwell assay (quantification in panel 3C). D, Correlations of HDAC1 with TPX2 and Snail in clinical CC tissues (n = 65) analyzed using immunohistochemistry (quantification in panel 6H). E, Ki67-positive cells and positive Snail and TPX2 protein expression in tumor tissues of nude mice treated with JSL-1 detected by immunohistochemistry (quantification in panel 7F). F, Wound healing ability of cells treated with JSL-1 assayed by scratch test (quantification in panel 8A).

**
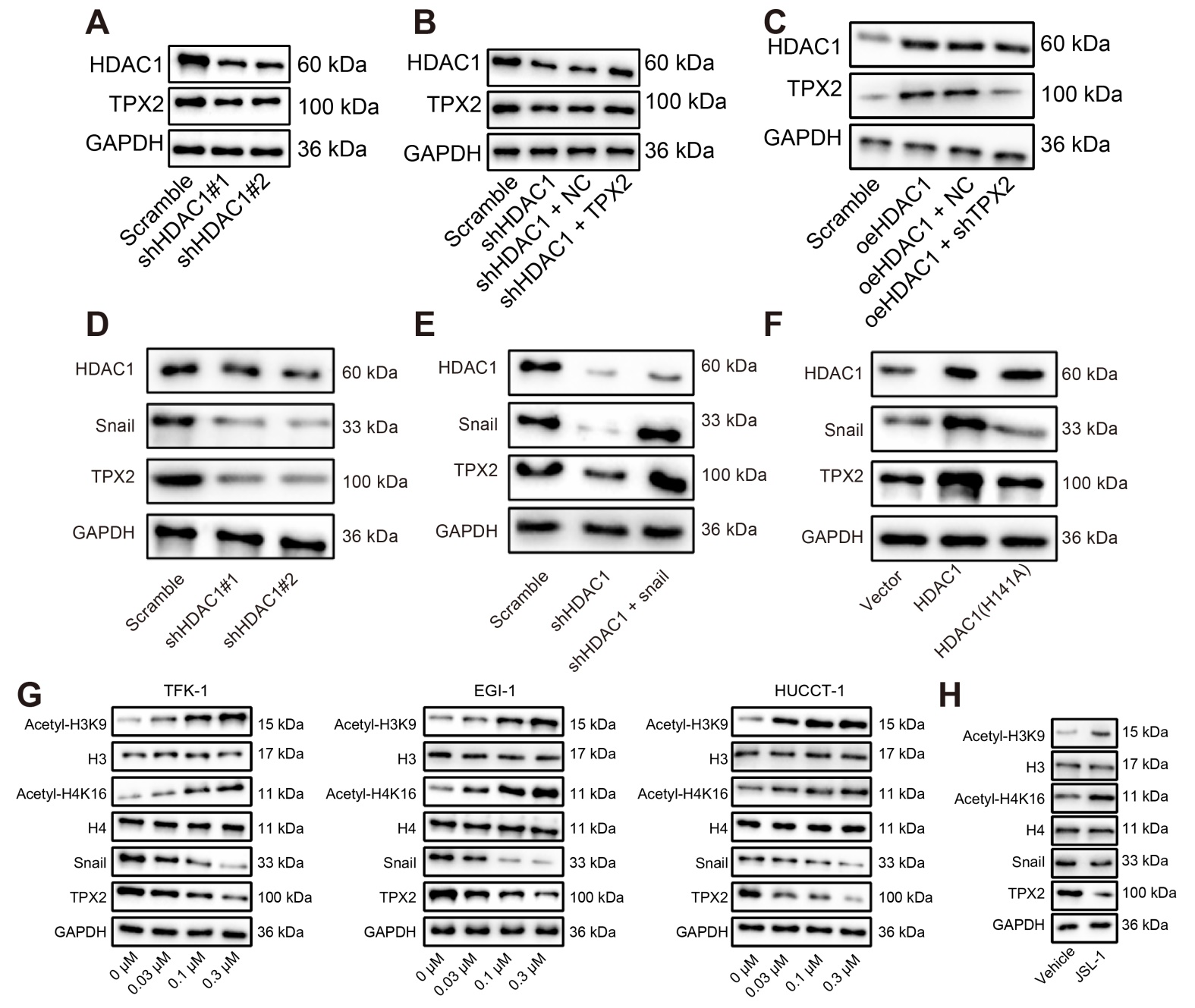
**

**Fig. S3** Representative images of immunoblots. A, Protein expression of HDAC1 and TPX2 in response to shHDAC1#1 and shHDAC1#2 (quantification in panel 4B). B, Protein expression of HDAC1 and TPX2 in response to HDAC1 silencing alone or combined with TPX2 overexpression (quantification in panel 4C). C, Protein expression of HDAC1 and TPX2 in response to HDAC1 overexpression alone or combined with TPX2 silencing (quantification in panel 4J). D, Protein expression of HDAC1, Snail, and TPX2 in response to shHDAC1#1 and shHDAC1#2 (quantification in panel 6A). E, Protein expression of HDAC1, Snail, and TPX2 in response to HDAC1 silencing alone or combined with snail overexpression (quantification in panel 6C). F, Protein expression of HDAC1, Snail, and TPX2 in response to HDAC1 overexpression or HDAC1 mutation (quantification in panel 6C). G, Acetylation level of H3K9 and H4K16 and expression of Snail and TPX2 proteins in TFK-1 cells upon JSL-1 treatment (quantification in panel 7A). H, The expression of Snail and TPX2 proteins and acetylation level of H3K9 and H4K16 in tumor tissues of nude mice after JSL-1 treatment measured by Western blot analysis (quantification in panel 7G).

**Supplementary table 1** Primer sequences for RT-qPCR

| Gene | Sequence |
| --- | --- |
| HDAC1 (human) | F: 5’-CTACTACGACGGGGATGTTGG-3’ |
|  | R: 5’-TGTTGCCAGAGACGAAGTGGAG-3’ |
| GAPDH (human) | F: 5’-ACCTGACCTGCCGTCTAGAA-3’ |
|  | R: 5’-TCCACCACCCTGTTGCTGTA-3’ |
| TPX2 (human) | F: 5’-ATGGAACTGGAGGGCTTTTTC-3’ |
|  | R: 5’-TGTTGTCAACTGGTTTCAAAGGT-3’ |
| Snail (human) | F: 5’-TCGGAAGCCTAACTACAGCGA-3’ |
|  | R: 5’-AGATGAGCATTGGCAGCGAG-3’ |

Note: RT-qPCR, reverse transcription quantitative polymerase chain reaction; F, forward; R, reverse; HDAC1, histone deacetylase 1; GAPDH, glyceraldehyde-3-phosphate dehydrogenase; TPX2, xenopus kinesin-like protein 2.
